# Rapid Quantitative Detection of *Brucella melitensis* by a Label-Free Impedance Immunosensor Based on a Gold Nanoparticle-Modified Screen-Printed Carbon Electrode

**DOI:** 10.3390/s130708551

**Published:** 2013-07-04

**Authors:** Haiyun Wu, Yueming Zuo, Chuanjin Cui, Wei Yang, Haili Ma, Xiaowen Wang

**Affiliations:** 1 College of Engineering, Shanxi Agriculture University, Taigu 030801, China; E-Mails: haiyunwu2013@163.com (H.W.); 200808doudou@sina.cn (W.Y.); 2 College of Electrical Engineering, Hebei United University, Tangshan 063009, China; E-Mail: cuichuanjin@163.com; 3 College of Animal Science and Technology, Shanxi Agriculture University, Taigu 030801, China; E-Mail: mahaili1718@126.com; 4 College of Food Science & Technology, Shanxi Agriculture University, Taigu 030801, China; E-Mail: wangx@sxau.edu.cn

**Keywords:** quantitative detection, *Brucella melitensis*, label-free immunosensor, screen-printed carbon electrode, gold nanoparticle

## Abstract

A rapid and simple method for quantitative monitoring of *Brucella melitensis* using electrochemical impedance spectroscopy (EIS) is reported for the first time. The label-free immunosensors were fabricated by immobilizing *Brucella melitensis* antibody on the surface of gold nanoparticle-modified screen-printed carbon electrodes (GNP-SPCEs). Cyclic voltammetry (CV) and EIS were used to characterize the *Brucella melitensis* antigen interaction on the surface of GNP-SPCEs with antibody. A general electronic equivalent model of an electrochemical cell was introduced for interpretation of the impedance components of the system. The results showed that the change in electron-transfer resistance (*R*_ct_) was significantly different due to the binding of *Brucella melitensis* cells. A linear relationship between the *R*_ct_ variation and logarithmic value of the cell concentration was found from 4 × 10^4^ to 4 × 10^6^ CFU/mL in pure culture. The label-free impedance biosensor was able to detect as low as 1 × 10^4^ and 4 × 10^5^ CFU/mL of *Brucella melitensis* in pure culture and milk samples, respectively, in less than 1.5 h. Moreover, a good selectivity *versus Escherichia coli* O157:H7 and *Staphylococcus aureus* cells was obtained for our developed immunosensor demonstrating its specificity towards only *Brucella melitensis*.

## 1. Introduction

Brucellosis is a worldwide bacterial zoonosis and an important cause for human suffering and economical losses. Brucellosis incidence increased sharply in China since 1995 [1]. About 43,623 cases were reported during 1999–2008 [2]. Brucellosis infections are almost invariably transmitted to people by direct or indirect contact with infected animals or their products. In particular, consumption of unpasteurized milk and dairy products is one of the most important sources of human infection [3].

The etiological agents of brucellosis are various *Brucella* species, including *Brucella abortus*, *Brucella melitensis*, *Brucella suis*, *Brucella neotomae*, *Brucella ovis*, and *Brucella canis*. In general, *Brucella melitensis* and *Brucella suis* are more virulent for humans than *Brucella abortus* and *Brucella canis*, although serious complications can occur with any species of *Brucella* [4]. Currently, Brucella isolation by bacterial culture is still a gold standard method that confirms the existence of the causative agent [5]. Other techniques such as complement fixation test (CFT), serum agglutination test (SAT), Rose Bengal plate test (RBT) and polymerase chain reaction (PCR) are used as supporting methods [3,6,7]. Regrettably, these methods have their limitations, such as labor and time-consuming, requiring complicated sample pretreatment and highly qualified personnel. Moreover, most of these procedures are only adapted for the qualitative or semiquantitative detection for *Brucella assays*, which do not meet the requirements for the rapid and accurate identification of brucellosis in less time that delays the introduction of efficient remedial measures. As a consequence, the development of rapid, inexpensive, and easy-to-use detection methodologies for *Brucella*, which can be used by untrained personnel, is vital.

One of the most promising novel techniques is the use of immunosensors. This technology makes use of immunochemicals as molecular recognition elements to construct self-contained devices. Due to high selectivity and sensitivity, immunosensors have been the subject of continued research and development in recent years. Several immunosensors for the detection of *Brucella* have been reported [5,8,9]. However, most of these sensors are label-dependent that require labeling of bio-molecules to convert the antibody/antigen interaction into detectable optical or electrochemical signals. In contrast label-free immunosensors have attractive advantages with respect to speed, cost, and simplicity of operation [10]. Hence development of label-free biosensors for the detection of *Brucella* directly in biological samples such as milk, serum, or urine, *etc.*, has attracted the considerable interest.

Label-free detection methodologies for *Brucella* using surface plasmon resonance (SPR) were reported [11]. The expense of the sensor materials or uncommon measuring instruments would limit out-of-laboratory applications for economic and fast screening. Fortunately, the impedance technique is yet another rapid and inexpensive alternative for label-free biosensors. Electrochemical impedance measurement devices are also suitable for mass fabrication and miniaturization. Traditionally, metal macro-sized metal rods or wires were used as electrodes immersed in a medium to measure the electrochemical response [8]. Due to recent developments in biosensor technology, the production of electrochemical transducers using screen-printed carbon electrodes (SPCEs) is well established. Electrochemical immunosensors based on SPCEs challenge the conventional electrochemical biosensors for fabrication, disposability and portability, which make them suitable for working with microvolumes and for decentralized assays (point of care tests) [12]. In particular, the coupling of screen-printed electrodes with metal nanoparticles (such as gold nanoparticles, GNPs) in electrochemical immunosensors has received considerable attention. Since GNPs have large specific surface areas and good bio-compatibility, the use of GNPs as versatile and efficient templates for the immobilization of biomolecules, such as antibodies, target cells or viruses, have been reported by many groups [13–16]. Many recent studies are focused on *Escherichia coli* and *Salmonella* detection with electrochemical techniques: impedimetric [17,18], amperometric [14,19] and capacitive [20] measurements, however, few studies were devoted to *Brucella melitensis* detection.

In this work, we demonstrated a disposable gold nanoparticle-modified screen-printed carbon electrode (GNP-SPCE)-based impedance immunosensor as a new approach for the rapid, simple and quantitative detection of *Brucella melitensis*. Gold nanoparticles on the electrode will result in large electrode surface area and easy attachment of antibody. The recognition of a monolayer of antibodies immobilised on an electrode by the specific antigens was monitored. Compared to routine immune diagnosis method, this label-free immunosensor is a relatively simple operation and low-cost, which can be used by untrained personnel. This establishes a novel approach for fast detecting *Brucella* organisms in a point of care real time situation.

## 2. Experimental Section

### 2.1. Reagents

Bovine serum albumin (BSA) and proteinase K were purchased from Roche Diagnostics GmbH (Mannheim, Germany). Triton X-100 was obtained from Amresco LLC (Solon, OH, USA). K_4_Fe(CN)_6_ K_3_Fe(CN)_6_, and KCl were purchased from Solarbio Science & Technology CO., Ltd. (Beijing, China). Brucellosis positive standard serum (1,000 IU/mL) was purchased from China Institute of Veterinary Drug Control (Beijing, China). A 1:25 dilution of the serum was prepared in PBS (10 mM, pH 7.4) before use. The buffers and solutions used in this study were prepared as follows: PBS buffer (10 mM, pH 7.4), blocking buffer (1% BSA in PBS buffer), and electrolyte solution (2.5 mM K_4_Fe(CN)_6_, 2.5 mM K_3_Fe(CN)_6_ and 0.1 M KCl in PBS buffer). All solutions were prepared with deionized water in a Heal Force water purification system (Smart Series, 18.2 MΩ·cm, Hong Kong, China).

### 2.2. Instruments

Scanning electron microscopy (SEM) images of electrode surface were obtained using a JEOL-JSM-6490LV scanning electron microscope (JEOL Ltd., Tokyo, Japan). EIS and cyclic voltammetry (CV) measurements were performed with a CHI 760C electrochemical station (CH Instruments, Shanghai Chenhua, Shanghai, China). All experiments were carried out at room temperature.

**Figure 1.**
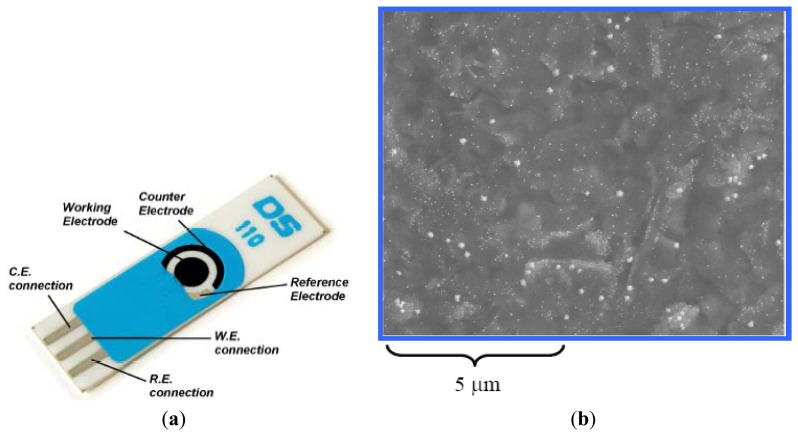
Images of the GNP-SPCE. (**a**) Photo of the electrode; (**b**) SEM image of working electrode. The figures originated from DropSens Inc. (Oviedo, Spain).

SPCEs functionalized with gold nanoparaticles on ceramic substrate (L 34 mm × W 10 mm × H 0.5 mm) were purchased from DropSens Inc. (Oviedo, Spain). The disposable electrode consisted of a GNP-carbon working electrode; a carbon counter electrode and a silver reference electrode (Figure 1).

### 2.3. Preparation of Microbial Sample

*Brucella melitensis* (4 × 10^10^ colony forming units (CFU)/mL) was purchased from China Institute of Veterinary Drug Control (Beijing, China). The other bacterial cultures used in this study, including heat-killed *Escherichia coli* O157:H7 cells (1.15 × 10^9^ CFU/mL) and *Staphylococcus aureus* strain (C56024), were obtained from Kirkegaard & Perry Laboratories, Inc. (Gaithersburg, MD, USA) and the Food Science and Technology College of Shanxi Agricultural University (Taigu, China), respectively. A 10-μL loop of *Staphylococcus aureus isolate* was grown in Luria-Bertani (LB) liquid culture at 37 °C for 24 h to make a stock culture. The stock cultures were serially diluted with PBS buffer. A conventional spread plating method was used for bacterial counts.

A whole pasteurized milk sample was purchased from a local supermarket. In analysis of real-life samples, the milk samples were spiked with different concentrations of *Brucella melitensis* cells. Next, the samples were treated by the method [14] to eliminate lipids and proteins. Briefly, proteinase K (0.25 mg) and 50 μL of 0.1% Triton X-100 were added to 100 μL samples of milk, and then incubated at 37 °C for 30 min. After incubation, 900 μL of 150 mM NaCl was added to the samples and the mixture was centrifuged at 12,000×g for 10 min. The lipids (top layer) and digested proteins of the milk were drawn off with a micropipette and the bacteria-containing pellets were collected. Finally, the pellets were resuspended in 100 μL of 150 mM NaCl and used for detection of bacteria.

### 2.4. Fabrication of the Immunosensors

Each working electrode of the GNP-SPCE was incubated with 15 μL dilute brucellosis positive standard serum for 30 min at 37 °C. The GNP-SPCE was then slowly placed in PBS buffer for 30 s, allowing the diffusion of unbound antibodies away from the electrode surface. After this, the GNP-SPCE was rinsed with deionized water two times and blew away residual drops with a stream of nitrogen. This was then followed by blocked each working electrode of the GNP-SPCE with 15 μL of blocking buffer for 60 min at 37 °C. Finally, they were slowly immersed in PBS buffer for 30 s and washed twice with deionized water. Then they were blew again with a stream of nitrogen and stored at room temperature in a clean box before use.

### 2.5. Measurement Procedure

A volume of 15 μL sample for test was applied to the immunosensor on the working electrode and incubated for 60 min at 37 °C. To remove nonspecifically bound cells, the sensors were then rinsed thoroughly with PBS and deionized water twice each and blew awhile with a stream of nitrogen.

For all CV and EIS measurements, 50 μL of electrolyte solution applied to the electrochemical reaction area of the GNP-SPCE immunosensor and incubated for 60 s at room temperature.

Potential of CV experiments was scanned from -1.2 to 1.2 V with a scan rate of 100 mV/s. All of current and potential data were recorded by the electrochemical station.

All EIS tests were conducted in an open circuit (the open circuit potential is 0.13 V). Nyquist diagrams (imaginary impedance *vs.* real impedance) were recorded from 0.1 Hz to 100 KHz with amplitude of 5 mV.

### 2.6. Data Analysis

EIS data were used for simulation of the immunosensor by the ZVIEW software. Seventy-two data points from each measured spectrum were automatically selected by the software for inputting into an equivalent circuit to generate a fitting spectrum. The difference in electron-transfer resistance before and after the bacterial cells binding to the sensor surface was taken as the signal produced by the immune reaction between immobilized antibodies and the cells. Besides, each experiment was repeated three times using three different GNP-SPCEs to test the reproducibility of the immunosensor. All data were expressed with mean signals and ±standard deviation (S.D.).

## 3. Results and Discussion

### 3.1. Electrochemical Characteristics of Brucella Melitensis Immunosensor

**Figure 2.**
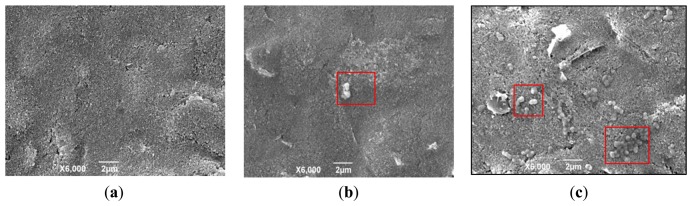
SEM images of the GNP-SPCE surface. (**a**) Clean surface; (**b**) Bacteria bound to surface from sample of 4 × 10^3^ CFU/mL; (**c**) Bacteria bound to surface from sample of 4 × 10^7^ CFU/mL.

On the GNP-SPCE surface, the GNPs were used to increase the electrode specific surface areas and greatly increase the amount of immobilized biomolecules. The results of *Brucella melitensis* cells bound to the surface of the GNP-modified SPCE with antibodies immobilized are shown by SEM images (Figure 2). Figure 2a shows the clean surface of the GNP-SPCE before used. Figure 2b,c shows the difference between a solution with low cell concentrations (4 × 10^3^ CFU/mL) and high cell concentrations (4 × 10^7^ CFU/mL). It can be seen clearly that the solution with low cell concentrations results in few bacteria being immobilized on the sensor surface, while the solution with high cell concentrations results in much more bacteria immobilized on the sensor surface. The *Brucella melitensis* cells were observed to be ellipsoidal (in the red square frame) that were interacted with antibodies on the GNP-SPCE surface by noncovalent bonds [21].

**Figure 3.**
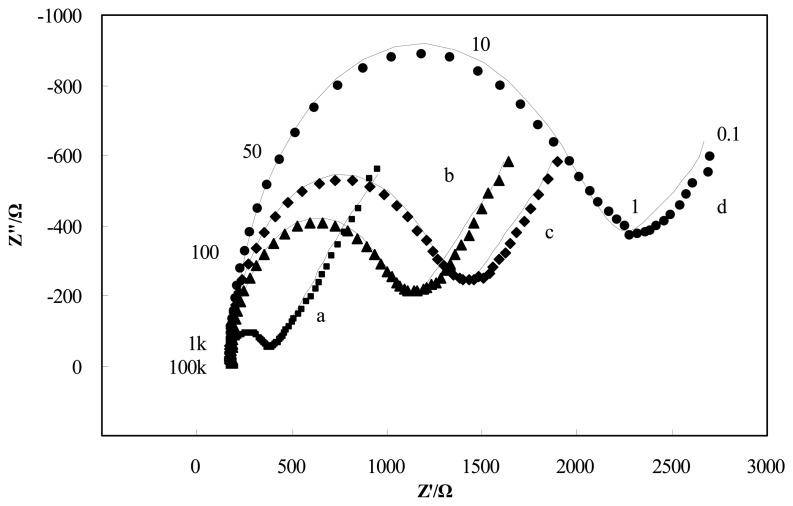
Nyquist diagrams and the corresponding simulated results (solid line) of GNP-SPCEs obtained under the following conditions: (**a**) at the bare GNP-SPCE; (**b**) after antibody immobilization; (**c**) after blocked with BSA; and (**d**) after *Brucella melitensis* cells (4 × 10^5^ CFU/mL) binding in the frequency range from 0.1 Hz to 100 kHz. A sinusoidal potential modulation of ±5 mV amplitude was superimposed on the open circuit potential (0.13 V). Electrolyte: 2.5 mM K_4_[Fe(CN)_6_] + 2.5mM K_3_[Fe(CN)_6_] + 0.1 M KCl + 10 mM PBS (pH = 7.4).

The adsorption of insulating materials on conductive supports is anticipated to alter the interfacial electron transfer features at the electrode surface [22]. Therefore, impedance experiments were used for confirmation of the stepwise changes of the *Brucella melitensis* immunosensor. Figure 3 depicts the Nyquist diagrams of GNP-SPCEs obtained under the following conditions: (a) at the bare GNP-SPCE; (b) after antibody immobilization; (c) after blocked with BSA; and (d) after *Brucella melitensis* cells (4 × 10^5^ CFU/mL) binding in the presence of [Fe(CN)_6_]^3-/4-^ as a redox probe. As shown in Figure 3, each of the impedance spectra includes a semicircle portion and a linear line portion, which corresponds to the electron transfer process and diffusion process respectively. The Nyquist diagrams can be simulated with an equivalent circuit which is given in Scheme I, where *R*_S_ is the resistance of the electrolyte solution, *CPE* is the constant phase element, *R*_ct_ is the electron-transfer resistance, and *W* is the Warburg element [23]. The impedance of *CPE* and *W* are given by the flowing equations [24]:

(1) $$Z_{\text{CPE}}=\frac{1}{Q\times {\left(\text{j}\omega \right)}^{a}}$$

(2) $$Z_{W}=R\times \text{ctnh}[{\left(jT\omega \right)}^{P}]/{\left(jT\omega \right)}^{P}$$

where *Q* and *α* are the characters of constant phase element. *R*, *T*, and *P* are the characteristic values of Warburg element. *R* is a resistance. *T* is equal to L^2^/D (L is the effective diffusion thickness, and D is the effective diffusion coefficient). *P is* an exponent. *ω* is the frequence of excitation sources and *j* is the imaginary unit. To use Equation (2), we set *P* = 0.5.

By fitting the electrochemical impedance spectra to the equivalent circuit, the simulated results are shown in Figure 3 (solid line) and the values of all electrical elements in the equivalent circuit are listed in Table 1. According to the Table 1, the electron-transfer resistance of a GNP-modified SPCE (Figure 3, curve a) is 204.6 Ω, which is very small compared with the values of *R*_ct_ for the other cases. It agrees with the fact that the electron transfer process on the GNP-modified SPCE surface is very fast, which is almost a diffusional limited electron transfer process. The immobilization of antibodies on the electrode surface occurred through direct physical adsorption of the molecules. Assembly of antibody on the GNP-SPCE surface (Figure 3, curve b) generated almost no change in *R*_S_ and the Warburg element values, but significant change in *R*_ct_ due to the immobilized insulating protein layer on the electrode surface introduced an electron-transfer barrier. The increased electron-transfer resistance in Nyquist plots after BSA blocking (Figure 3, curve c) was because BSA has blocked the free sites on the sensor surface, leaving only the available recognition sites for antigen binding. Similarly, after the treatment of the antigen–antibody interaction was made (Figure 3, curve d), the increment of *R*_ct_ became the most significant among all the elements in the table and the increment rate increased quite markedly. From these results, it can be summarized that electrochemical impedance spectroscopy is capable of monitoring the change in electron transfer resistance resulting from the immobilization of antibodies and the binding of *Brucella melitensis* cells.

**Figure 4.**
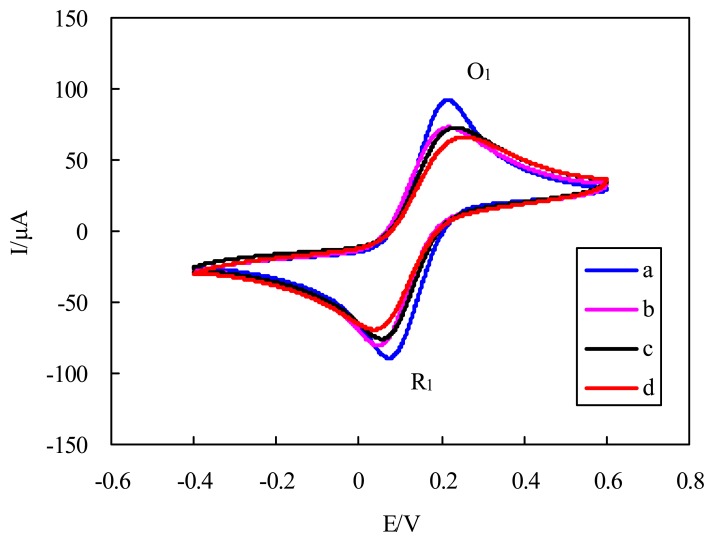
Cyclic voltammograms of the GNP-SPCEs obtained under the following conditions: (**a**) at the bare GNP-modified SPCE; (**b**) after antibody immobilization; (**c**) after BSA blocking; and (**d**) after cell binding (4 × 10^5^ CFU/mL) in the solution of 2.5 mM K_4_[Fe(CN)_6_] + 2.5mM K_3_[Fe(CN)_6_] + 0.1 M KCl + 10 mM PBS (pH = 7.4). Scan rate, 100 mV/s. The reduction peak: R_1_. The oxidation peak: O_1_.

CV experiments were used to further confirm the surface changes for the attachment of antibodies and antigens on the immunosensor. Theoretically, the changes in peak current and the separation of peak potentials in voltammograms at different electrode surfaces are related to the electron-transfer rate constant, and thus, the electron-transfer resistance [25]. Figure 4 shows the cyclic voltammograms of the GNP-SPCEs obtained under the following conditions: (a) at the bare GNP-modified SPCE; (b) after antibody immobilization; (c) after BSA blocking; and (d) after cell binding (4 × 10^5^ CFU/mL) in PBS solution containing 2.5 mM [Fe(CN)_6_]^3-/4-^(1:1 mole ratio) and 0.1 M KCl. When the electrode was immobilized with antibodies, a decrease in peak current (from 100.1 to 77.4 μA) and an increase in the separation of peak potentials (from 142 to 170 mV) were observed. After the electrode was blocked with BSA, the electron-transfer of the electrode was measured to decrease further from 77.4 to 74.1 μA. Especially, a decrease of 20.2% in peak current (from 74.1 to 59.1 μA) was clearly observed after the antigen–antibody interaction. The cell binding also resulted in an increase of 22.2% in the separation of peak potentials (from 180 to 220 mV). The increase in the separation of the peak potentials and decrease in peak current after the different electrode processing indicate that the immobilization of antibody and the binding of cells perturb the electron transfer rate, which are in good agreement with the increase in the electron transfer resistance observed in the impedance spectra.

### 3.2. Detection Limits and Specificity of Brucella Melitensis Immunosensor

**Figure 5.**
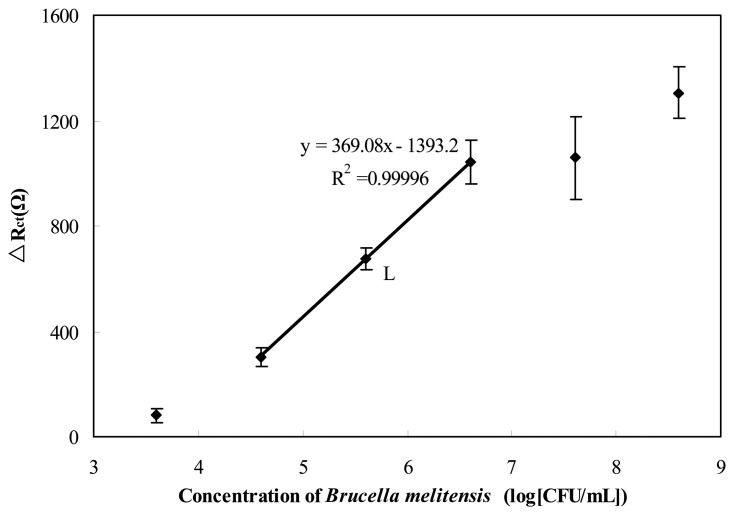
The Δ*R*_ct_ due to the different concentrations of *Brucella melitensis* cells detected by the GNP–SPCE immunosensor. Δ*R*_ct_ was calculated by *R*_ct(Ab-Ag)_ - *R*_ct(Ab)_, where *R*_ct(Ab-Ag)_ is the value of the electron transfer resistance after antigen binding to antibody, *R*_ct(Ab)_ is the value of the electron transfer resistance after BSA blocking. Each value of *R*_ct_ was derived from three independent measurements.

*Brucella melitensis* concentrations, from 4 × 10^3^ to 4 × 10^8^ CFU/mL were prepared by serial dilution in PBS buffer. A volume of 15 μL sample of each bacterial suspension was taken for immunosensing to evaluate the detection range by calculation of the electron-transfer resistance change before and after cells binding (Δ*R*_ct_). Figure 5 shows the derived calibration plot that corresponds to the electron-transfer resistance changes at the sensing interface with different concentrations of cell. A linear relationship between the value of Δ*R*_ct_ and logarithmic value of the cell concentration was found from 4 × 10^4^ to 4 × 10^6^ CFU/mL. The detection limit of the immunosensor was approximately 1 × 10^4^ CFU/mL in PBS(the limit of detection was calculated as the concentration yielding a signal equal to three times the standard deviation of the blank (intercept) divided by the slope). The total test process, including binding, washing, and sensing, took about 1.5 h for each test.

**Figure 6.**
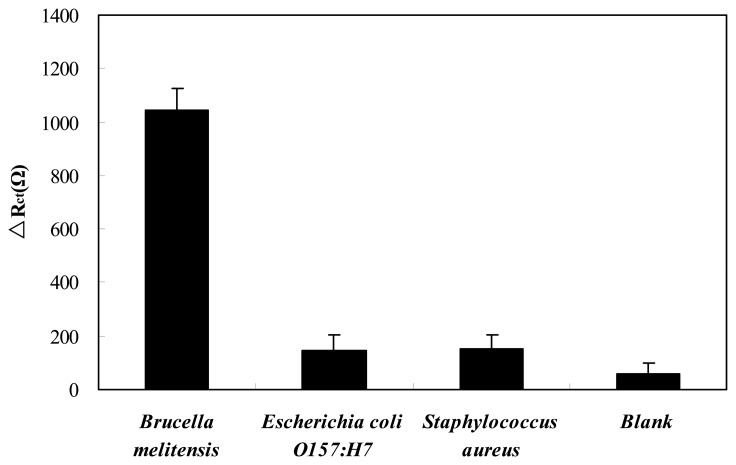
Specificity of the GNP-SPCE immunosenor for *Brucella melitensis*.

The cross-reactivity for antibodies is an important concern in immunoassy. To assess the specificity of the GNP–SPCE immunosenor for *Brucella melitensis*, interference study was conducted with *Escherichia coli* O157:H7 and *Staphylococcus aureus* at a concentration around 4 × 10^6^ CFU/mL as depicted in Figure 6. It can be seen that the value of Δ*R*_ct_ for *Brucella melitensis* is significantly higher than the values obtained in blank. While the values of Δ*R*_ct_ for the bacteria other than *Brucella melitensis* are similar to blank. Thereby the cross-reactivity of the *Brucella melitensis* antibodies to *Escherichia coli* O157:H7 and *Staphylococcus aureus* is negligible. The results indicate that the immuosensor has good specificity for *Brucella melitensi*s detection.

### 3.3. Detection of Brucella Melitensis in Real-Life Samples

**Figure 7.**
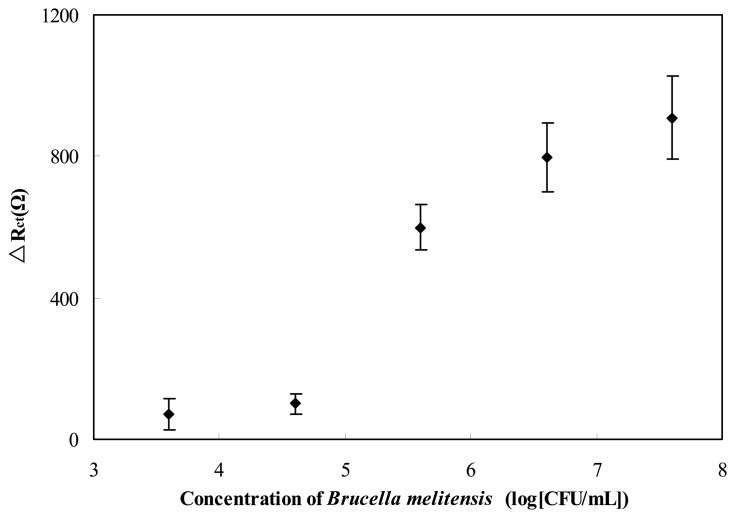
Detection of *Brucella melitensis* in milk samples. The mean of each Δ*R*_ct_ was calculated from three independent measurements of GNP-SPCE immunosensors.

Brucellosis, especially that arising from *Brucella melitensis*, is often foodborne, and unpasteurized milk and dairy products are common vehicles of transmission. In order to test the sensitivity of the immunosensor, different concentrations of *Brucella melitensis* cells were spiked with milk to simulate the real-life milk sample. A volume of 15 μL sample of each bacterial suspension was evaluated with this developed immunosensor and the analysis results are reported in Figure 7. Statistical significance of mean differences between concentrations was evaluated by a one-way ANOVA and t test to a significance of 95% (p < 0.05). The statistical analysis shows that the lower detection limit was 4 × 10^5^ CFU/mL. These results reveal that this immunosensor is capable of *Brucella melitensis* detection present in milk. But how to use this technique in reality still need to be studied further.

## 4. Conclusions

A label-free impedance immunosensor for *Brucella melitensis* detection was developed based on GNP-SPCE. The disposable GNP-SPCE was used for sensing by electrochemical impedance spectroscopy (EIS) in the presence of [Fe(CN)_6_]^3-/4-^]. A general electronic equivalent model was introduced for modeling the performance of the immunosensor. Among these impedance components, the greatest change was found in electron-transfer resistance due to the cell binding. The label-free impedance biosensor showed linearity from 4 × 10^4^ to 4 × 10^6^ CFU/mL with a detection limit of 1 × 10^4^ CFU/mL in pure culture. When testing real-life samples, the biosensor was able to detect as low as 4 × 10^5^ CFU/mL of *Brucella melitensis* in milk samples in less than 1.5 h. Interference from other bacteria was eliminated by the use of specific antibodies. This biosensing method uses a low-cost disposable electrode. It is hand-held operation, and no complex pretreatment is needed, thus would allow in the future the development of easy-to-use and portable devices for the rapid monitoring of *Brucella melitensis*.

## Figures and Tables

**Scheme I.**
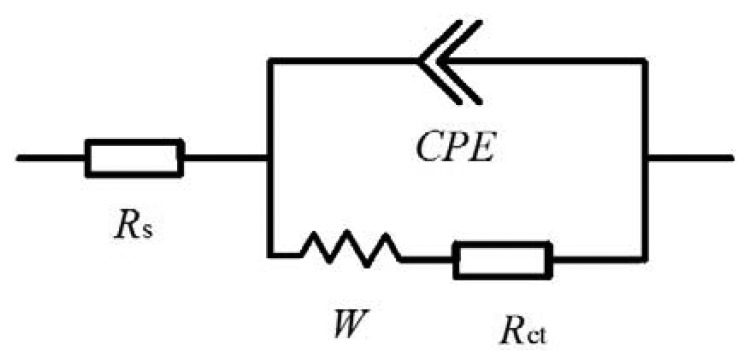
Equivalent circuit for Nyquist plots. *R*_S_, solution resistance; *CPE*, constant phase element; *R*_ct_, electron-transfer resistance; *W*, Warburg element due to diffusion of the redox couple ([Fe(CN)_6_]^4-^/[Fe(CN)_6_]^3-^) to the interface from the bulk of the electrolyte.

**Table 1.** Simulated values of all elements in the equivalent circuit and percentage of their changes ^a^.

**Circuit Elements**	***R***_S_ [**Ω**]	***CPE***		***R***_ct_ [**Ω**]	***W***	
	
		***Q*** [**μ;F·s**^−(1−α)^]	**α**		***W*_R_** [**Ω**]	***W*_T_** [**s]**
GNP-modified SPCE	167.60 ± 0.78	2.72 ± 0.25	0.92 ± 0.01	204.60 ± 2.25	2224 ± 174.91	9.79 ± 1.62
After antibody immobilization	175.90 ± 0.60	5.13 ± 0.18	0.97 ± 0.00	849.60 ± 6.02	2080 ± 169.49	7.52 ± 1.37
Increment rate	4.95%	88.40%	5.25%	315.25%	−6.47%	−23.19%
After antibody immobilization	175.90 ± 0.60	5.13 ± 0.18	0.97 ± 0.00	849.60 ± 6.02	2080 ± 169.49	7.52 ± 1.37
After BSA blocking	172.70 ± 0.58	5.32 ± 0.14	0.96 ± 0.00	1111 ± 7.25	2097 ± 195.05	7.49 ± 1.57
Increment rate	−1.82%	3.62%	−0.15%	30.77%	0.82%	−0.37%
After BSA blocking	172.70 ± 0.58	5.32 ± 0.14	0.96 ± 0.00	1111 ± 7.25	2097 ± 195.05	7.49 ± 1.57
After *Brucella melitensis* binding	175.40 ± 0.61	6.28 ± 0.13	0.96 ± 0.00	1788 ± 12.98	2124 ± 246.22	6.74 ± 1.77
Increment rate	1.56%	18.09%	−0.57%	60.94%	1.29%	−10.01%

a Numbers of data for simulation,72; *Brucella melitensis*, 4 × 10^5^ CFU/mL.
